# Pro-inflammatory diet and depressive symptoms in the healthcare setting

**DOI:** 10.1186/s12888-022-03771-z

**Published:** 2022-02-17

**Authors:** Rachel Belliveau, Sydney Horton, Courtney Hereford, Lance Ridpath, Robert Foster, Emily Boothe

**Affiliations:** 1 University of North Carolina Health Care, 2201 S Sterling St, Morganton, NC USA; 2 University of Texas Health Science Center at Tyler, 11937 US-271, Tyler, TX USA; 3 Center for Rural and Community Health, West Virginia School of Osteopathic Medicine, 400 N Lee St, Lewisburg, WV USA; 4 Institutional Research Assessment Educational Development, West Virginia School of Osteopathic Medicine, 400 N Lee St, Lewisburg, WV USA; 5 West Virginia School of Osteopathic Medicine, 400 N Lee St, Lewisburg, WV USA; 6 Department of Psychiatry, Princeton Community Hospital, 122 12th St, Princeton, WV USA

**Keywords:** Depression, Nutrition, Empirical dietary inflammatory index (EDII), Patient health questionnaire (PHQ-9), Healthcare setting

## Abstract

**Background:**

Depression is a highly prevalent disease affecting more than 350 million people and has recently been associated with low-grade chronic inflammation. The role of diet in the development of a pro-inflammatory state was demonstrated in a recent study that found a high Empirical Dietary Inflammatory Index (EDII) score was associated with increased concentrations of circulating plasma inflammatory markers. It is becoming increasingly clear that diet and depression are linked, but the relationship itself has not been determined with absolute certainty. Pharmacologic and device assisted therapies are considered our most advanced treatments for major depressive disorder, though numerous studies suggest that they are not sufficient. Exploring the relationship of a pro-inflammatory diet and depressive symptoms is crucial for a holistic, evidenced-based approach to treating depression.

**Methods:**

Our study investigated this association using the EDII to evaluate the inflammatory potential of diet and Patient Health Questionnaire-9 (PHQ-9) to score depression among healthcare personnel. Results from 631 participants were collected for analysis using REDCap software.

**Results:**

High PHQ-9 scores and high EDII scores were significantly correlated (*F* = 18.32, *p* < 0.0001) even when accounting for gender, psychiatric diagnosis, physical exercise, and spiritual exercise.

**Conclusions:**

Our findings suggest that anti-inflammatory diets can play a key role in the treatment of depression.

## Background

Depression is a highly prevalent disease affecting more than 350 million people worldwide and continues to be a leading cause of global disability [1]. The limitations with current treatment regimens have prompted the investigation of lifestyle modifications as alternative or adjunctive treatment. Recent studies of current treatments have not proven superior to placebo. For example, a meta-analysis of randomized, double-blinded, placebo-controlled studies found that escitalopram and repetitive transcranial magnetic stimulation trials both had significant responses to placebo [2, 3]. Similarly, a meta-analysis from Khan et al. found only a 51% depressive symptom reduction in published antidepressant data compared to 38% symptom reduction seen in the placebo data [4]. Those with chronic depression demonstrated a high rate of relapse within one year and no significant difference between fluoxetine and placebo groups [5]. Alarmingly, Food and Drug Administration clinical trial data suggests no significant difference in the incidence of suicide attempts and suicide rates between placebo and antidepressant drug-treated groups [6]. Pharmacologic and device assisted therapies are considered our most advanced treatments for symptoms of depression but it appears that they are not sufficient.

Growing evidence suggests that low-grade chronic inflammation may play a role in the development of depression [2, 7–9]. The symptoms of depression and fatigue are both associated with increased inflammation of the immune and nervous systems, and both depression and chronic stress involve persistent activation of the hypothalamic-pituitary-adrenal (HPA) axis [10, 11]. Pro-inflammatory cytokines, such as interleukins and tumor necrosis factor alpha (TNF-α), appear to be driving this activation by prolonging the secretion of cortisol, both by direct stimulation of the HPA axis and indirectly increasing sensitivity of glucocorticoid receptors [10]. Thirty-two percent of patients with major depressive disorder failed to suppress cortisol after dexamethasone administration [12]. Galecki et al. found similarly increased concentrations of inflammatory interleukins occurred in both the first incident of depression and in recurrent depression, suggesting dysregulation of pro-inflammatory cytokines as an underlying mechanism. This can have profound negative effects on brain structures such as the hippocampus, amygdala, and prefrontal cortex [10]. Lamers et al. not only found that current cases of major depressive disorder have elevated levels of the inflammatory marker interleukin-6 (IL-6), but that IL-6 is also elevated at two- and six-year follow-up [13]. Similarly, a meta-analysis by Valkanova et al. and Osimo et al. found that those with depression have higher levels of inflammatory markers such as C-reactive protein (CRP) and interleukin-12 (IL-12) [14, 15]. Studies such as these question whether modifiable factors could be driving this pro-inflammatory state and have sparked intrigue with the dietary-mental health relationship.

It has been established that a diet high in sugar, refined flour, saturated fats, and red and processed meats can activate inflammation. Such a diet has been linked to chronic conditions including cardiovascular disease, diabetes, cancers, and neurocognitive disorders [16]. Recently, this pro-inflammatory dietary pattern has been further associated with an increased risk of depression in adults [17, 18]. Neurotransmitters require sufficient amino acids, minerals, and vitamins to function properly, which may explain why certain nutritional deficiencies have been correlated with increased risk of depression [17]. The Mediterranean Diet, which consists of whole grains, vegetables, and fruits, with minimal dairy and meat, is recommended for an array of diseases due to its optimal nutritional balance [17]. The regular consumption of anti-inflammatory foods such as vegetables, fruits, and whole grains has been shown to reduce the risk for depression [19–21]. Although dietary intervention is not a standard of care in the treatment of depression, the evidence of its potential warrants further consideration.

Studies exploring the link between pro-inflammatory diet and depression have been on the rise in recent years. The Dietary Inflammatory Index (DII), validated in previous depression studies, correlates with elevated inflammatory markers such as CRP, IL-6 and TNF-α [22]. However, it may be too cumbersome for everyday clinicians to use, as it relies on detailed daily nutrient intake. The Empirical Dietary Inflammatory Index (EDII) is a new tool that has been validated in recent years [22]. While the DII is based on forty-five dietary factors known to increase inflammatory markers, mostly nutrient based, the EDII focuses on weekly consumption of sixteen net pro- or anti-inflammatory food groups. A study comparing the DII and EDII concluded that both indices can significantly predict elevation of plasma inflammatory markers through dietary evaluation, with the EDII proving superior [23]. To our knowledge, the EDII has not been used in any studies evaluating the relationship between depressive symptoms and a pro-inflammatory diet. Therefore, the aim of this study is to investigate the relationship between depressive symptoms and a pro-inflammatory diet using the EDII. We hypothesize that high EDII scores will be associated with greater incidence of depressive symptoms.

## Methods

Study participants were healthcare associates affiliated with Princeton Community Hospital (PCH) and/or West Virginia School of Osteopathic Medicine (WVSOM). This study was approved by electronic vote by the Institutional Review Board at PCH on October 1, 2020, in agreement with the Office of Student Research and Sponsored Programs at WVSOM. Designated representatives at PCH and WVSOM distributed the electronic survey link via institutional email on October 21, 2020. Participants voluntarily enrolled into the anonymous, electronic survey using REDCap software. Electronic informed consent was obtained from all individual study participants through an initial cover page read prior to opting into the survey.

Participants completing the study survey were redirected to a new and unlinked survey with the opportunity to voluntarily enter a random drawing for a $25.00 or $100.00 gift certificate in recognition of their time. Two follow-up survey invitations were disseminated to the study population at one- and two-week follow-up, with study closure on November 12, 2020. The survey included the EDII and Patient Health Questionnaire-9 (PHQ-9). Demographic survey questions included participant age range, gender, primary healthcare role, previous psychiatric diagnosis, current psychotropic medication use, typical weekly physical exercise, weekly meditation or mindfulness, and spiritual exercise or practice. Participation in regular meditation, mindfulness, and spiritual practices were included as participation in these activities has been shown to reduce symptoms in patients with depressive disorders in numerous studies [24]. Gender information was collected over biological sex to ensure inclusivity.

The EDII is a food frequency score that estimates the effect of diet on circulating plasma inflammatory biomarkers [25]. The EDII is scored using weekly consumption of eight pro-inflammatory and eight anti-inflammatory foods. The pro-inflammatory foods are red meats, processed meats, organ meats (i.e. liver, kidney, brain, heart), non-oily fish (i.e. white fish), eggs, sugar-sweetened beverages (i.e. soda), tomatoes, and refined grains (bread or pasta and white rice). The anti-inflammatory foods are leafy green vegetables, dark yellow vegetables (i.e. carrots, pumpkins, sweet potatoes, winter squash), fruit juice, oily fish (i.e. salmon, sardines), coffee, tea, wine, and beer or other alcoholic beverages [22, 25].

The EDII is scored using the Mediterranean Diet Pyramid based on weekly frequency of consumption [25]. Each pro-inflammatory food receives a score of 0, 1, or 2 points. Each anti-inflammatory food receives a score of 0, − 1, or − 2 points. Total scores range from − 16, indicating very low inflammatory potential of diet, to + 16, indicating a very high inflammatory potential of diet. The score can be further categorized into low inflammatory potential of diet at scores of − 9 to − 2, moderate at scores of − 1 to + 1, and high at scores of + 2 to + 10 [22, 25].

The PHQ-9 is a nine-item questionnaire that correlates the frequency of depressive symptoms, as are listed in Fourth Edition of the Diagnostic and Statistical Manual of Mental Disorders, to the severity of depression [26]. Each question is scored from 0 (not at all) to 3 (nearly every day). Total score ranges from 0–4 indicate absent or minimal depression, 5–9 indicate mild depression, 10–14 indicate moderate depression, 15–19 indicate moderately severe depression, and 20–27 indicate severe depression [27].

## Results

Statistical analyses were performed using SAS v9.4 software. Results from 631 participants were pooled (Table 1). The PHQ-9 score, transformed to be normally distributed using a square root transformation, was compared to the EDII Score and four covariates of gender, previous psychiatric diagnosis, physical exercise frequency, and spiritual exercise frequency to create a general linear model (Table 2). High PHQ-9 scores and high EDII scores occur concomitantly (*F* = 18.32, *p* < 0.0001) even when accounting for gender, psychiatric diagnosis, physical exercise, and spiritual exercise.

**Table 1:** Stratification of participant demographic data. Healthcare personnel were recruited from Princeton Community Hospital and West Virginia School of Osteopathic Medicine

Variable	Level	N	Mean	SD	Min	Max
*Previous psychiatric diagnosis*	No	448	5.71	5.01	0	27
	Yes	183	9.69	6.07	0	27
*Currently taking psychotropic medication*	No	492	6.11	5.22	0	27
	Yes	139	9.55	6.21	0	27
*Typical weekly physical exercise*	Less than once per week	172	8.73	5.94	0	27
	Once per week	101	6.62	5.56	0	27
	2–3 times per week	218	6.31	5.26	0	27
	Daily	140	5.62	5.33	0	27
*Typical weekly meditation/mindfulness exercise*	Less than once per week	367	7.54	5.85	0	27
	Once per week	80	6.56	5.07	0	20
	2–3 times per week	59	5.15	4.48	0	19
	Daily for less than 20 min	95	5.87	5.28	0	20
	Daily for greater than 20 min	30	5.87	6.33	0	26
*Typical weekly spiritual exercise*	None	174	7.39	5.68	0	26
	Less than once per week	132	7.67	5.17	0	23
	Once per week	93	7.08	6.06	0	27
	Daily	232	5.94	5.57	0	27
*Gender*	Female	471	7.35	5.75	0	27
	Male	157	5.37	5.02	0	26
	Non-binary/other	3	8.33	3.21	6	12
*Primary Role*	Faculty, administrator, or staff without direct patient care	188	6.5	5.65	0	27
	Physician or healthcare provider with direct patient care	163	7.61	5.66	0	23
	Student (DO, MD, NP, PA)	280	6.68	5.58	0	27

Key: *N* population size, *SD* standard deviation, *Min* minimum, *Max* maximum

**Table 2:** The transformed PHQ-9 score compared to the EDII Score and four covariates of gender, previous psychiatric diagnosis, physical exercise frequency, and spiritual exercise frequency to create a general linear model. High PHQ-9 scores and high EDII scores were significantly correlated even when accounting for gender, psychiatric diagnosis, physical exercise, and spiritual exercise

Source of Variation	df	Type III SS	MS	F	p	Sig.
*PHQ9 Score (transformed)*						
*Gender*	2	17.71	8.86	8.09	0.0003	**
*Previous Psych Diagnosis*	1	64.35	64.35	58.79	<.0001	**
*Physical Exercise Frequency*	3	15.79	5.26	4.81	0.0026	**
*Spiritual Exercise Frequency*	3	29.25	9.75	8.91	<.0001	**
*EDII Score*	1	20.05	20.05	18.32	<.0001	**
*Error*	615	673.21	1.09			
*Total*	625	820.36				

Key: *df* degrees of freedom, *SS* sum of squares, *MS* mean of squares, *F* F-ratio, *p p*-value, ** = statistically significant finding

Using Spearman’s correlation, several components of the EDII emerge as items of special interest (Table 3). Eating fewer leafy greens was correlated to higher PHQ-9 scores (r = − 0.14, *p* = 0.0003, *n* = 631), as were consuming greater amounts of bread or pasta (r = 0.15, *p* < 0.0001, *n* = 631), sugar sweetened beverages (r = 0.22, *p* < 0.0001, *n* = 631), and processed meats (r = 0.15, *p* = 0.0002, *n* = 631). The correlation between higher amounts of processed meats (greater than seven times per week) was most significantly linked to higher PHQ-9 scores when compared to those who consume processed meats fewer than two times per week.

**Table 3:** EDII score compared to PHQ-9 score. Low EDII score correlates to a lower PHQ-9 score; high EDII score correlates to a higher PHQ-9 score

	PHQ-9 Score		
EDII Score	N	EDII Score	N
*Low (−12 to − 2)*	166	*Low (−12 to − 2)*	166
*Moderate (−1 to + 1)*	306	*Moderate (− 1 to + 1)*	306
*High (+ 2 to + 10)*	159	*High (+ 2 to + 10)*	159

Key: *N* population, *SD* standard deviation

## Discussion

This cross-sectional study demonstrates that when controlling for four key demographic and lifestyle factors (gender, previous psychiatric diagnosis, physical exercise frequency, and spiritual exercise frequency), a pro-inflammatory EDII score correlates to increased depressive symptoms. These findings indicate that the EDII was able to produce results commensurate with other inflammatory diet and depression studies. These results also support the growing evidence that diet modification could prove to be an important tool in the treatment of depressive symptoms.

In one of the first studies of its kind, Bergmans and Malecki used the DII to investigate the relationship between depression and inflammatory diet. They found that a high DII score is associated with two times higher odds of depression in adults [8]. Similarly, Phillips et al. found that a high DII score increased the risk for depression among adults [2]. In meta-analyses, Tolkien et al. and Wang et al. found that individuals consuming a pro-inflammatory diet were at increased risk of developing depression [28, 29]. It becomes increasingly clear that diet and depression are linked, but the relationship itself has not been determined with absolute certainty. A diet that consists mainly of sugars, trans and saturated fats, and refined carbohydrates is a common finding among those with depression, and some studies have suggested that depression itself can alter the food choices we make [17].

In recent years there has been an implication of the gut microbiome in depression. It is suggested that psychological stressors create an imbalance of bacteria, which leads to intestinal permeability, allowing bacteria to enter the bloodstream. This triggers a production of pro-inflammatory cytokines, activating the HPA axis and crossing the blood-brain barrier to produce an inflammatory response [30]. If the pathological changes come first, this could create a viscous cycle of depression fueling inflammatory diet choices, and inflammatory diet fueling depressive symptoms in turn.

It is of note that a high DII score appears to put women at increased risk for recurrent depression over a five-year period [31]. Similarly, a recent study found that a pro-inflammatory diet at baseline increased the risk for depressive symptoms over a five-year period, especially among women [7]. It is important to make the connection between these studies and Lamers et al., who observed the continued elevation of IL-6 in those with depression at six-year follow-up [13]. It appears that some individuals with chronic depression remain in a pro-inflammatory state. With diet being one of the common denominators, it is logical to investigate whether dietary intervention can mitigate the inflammatory cycle.

A recent interventional study has shown that adherence to the Mediterranean Diet for three months can lead to a reduction in depressive symptoms and improvement in quality of life [32]. These results remained consistent at six-month follow-up. Parletta et al. also measured omega-3 and omega-6 fatty acid levels in participants. They found that improvement in depressive symptoms was associated with a deceased ratio of omega-6 to omega-3 fatty acids [32]. The Mediterranean Diet is rich in docosahexaenoic acid and eicosapentaenoic acid, two omega-3 fatty acids that act on brain structure and function. They are not only considered anti-inflammatory, but they play a role in the neurotransmission of serotonin and dopamine [9]. In comparison, omega-6 fatty acids are high in the traditional processed Western Diet and are considered pro-inflammatory [32]. Perhaps unsurprisingly, the Mediterranean Diet has been shown to positively alter the gut microbiome and increase its diversity [9]. These findings suggest that implementation of an anti-inflammatory diet could be key in breaking the depression-inflammation cycle. Further study is important to determine whether an anti-inflammatory diet can prevent depression altogether.

The main strength of this study was demonstrating that the EDII can correlate to depressive symptoms. Previous dietary indexes, such as the DII, have been largely unusable to the everyday clinician. With the DII’s comprisal of forty-five different factors, most of which are nutrients, it would be quite challenging to administer and score within a standard appointment time. Even with a DII score, clinicians may not discern diet modification based on individual nutrients alone. On the contrary, the EDII was designed for simplicity of use. With the focus on sixteen pro- and anti-inflammatory food groups, the tool can be easily self-administered by the patient and results calculated in minutes. The EDII may prove to be a useful assessment tool in the treatment of depression as it requires minimal appointment time and provides a clear focus for anti-inflammatory diet changes. It could also be revisited periodically to assess for improvements in inflammatory score.

There are multiple limitations with this study. First, the entirety of this study was administered through self-reported questionnaires with potential for recall bias among participants. Second, this study did not include a twenty-four-hour recall for the EDII portion of the survey. Although the twenty-four-hour recall has its own limitations, it is commonly used in dietary studies to obtain a more accurate dietary pattern among individuals [8]. A study validating a food frequency questionnaire using two twenty-four-hour recalls found moderate to high correlations for most food groups, with only cooked vegetables and pizza showing insignificant correlation. The proportion of participants classified into the same or adjacent quartile of consumption varied from 68% in cooked vegetables to 94% in coffee [33]. Thus, food frequency questionnaires alone prove to be valid assessment tools. Third, specific medical co-morbidities and body mass index information were not included as covariates.

There should be caution in applying the results of this study to the general population. The participants were only those associated with two specific healthcare settings, including physicians, nurses, and medical students who may experience higher daily stress than the general population. It is of note that this study was conducted during the Severe Acute Respiratory Syndrome Coronavirus 2 Pandemic, potentially inflating participants’ depressive symptoms. In a recent umbrella review of meta-analyses, approximately one in four healthcare workers reported depression and anxiety during the pandemic [34]. Respondents also skewed more heavily towards female gender (*n* = 471 female vs. *n* = 157 male vs. *n* = 3 non-binary/other). Finally, this is a cross-sectional study, and therefore only a correlation between pro-inflammatory EDII score and depression can be interpreted. As mentioned above, reverse causation should be considered, as some studies have indicated that stress and depression may lead to pro-inflammatory food choices [5].

## Conclusions

This study demonstrates a significant correlation between pro-inflammatory diet, as measured by EDII score, and depressive symptoms. The EDII, which has not been used in inflammatory diet and depression studies previously, was able to produce results consistent with similar studies. The findings of this study further reinforce the potential for anti-inflammatory diet in the prevention and treatment of depression. However, for clinicians to reliably utilize the EDII in the evaluation and treatment of depression, employing the EDII in dietary intervention is necessitated.

## Data Availability

All data generated or analyzed during this study are included in this published article.

## Abbreviations

CRP: C-Reactive Protein
DII: Dietary Inflammatory Index
EDII: Empirical Dietary Inflammatory Index
HPA axis: Hypothalamic-pituitary-adrenal axis
IL-6: Interleuken-6
IL-12: Interleuken-12
PCH: Princeton Community Hospital
PHQ-9: Patient Health Questionnaire-9
TNF-α: Tumor necrosis factor alpha
WVSOM: West Virginia School of Osteopathic Medicine

## References

[1] Marcus M, Yasamy MT, Ommeren MV, Chisholm D, Saxena S, Yasamy MT, m, V.O., Ommeren, M.V., & Chrisholm, D. (2012). Depression: a global public health concern. WHO Department of mental health and substance abuse.

[2] Phillips CM, Shivappa N, Hébert JR, Perry IJ (2018). Dietary inflammatory index and mental health: a cross-sectional analysis of the relationship with depressive symptoms, anxiety and well-being in adults. Clin Nutr.

[3] Brunoni A, Lopes M, Kaptchuk T, Fregni F (2009). Placebo response of non-pharmacological and pharmacological trials in major depression: a systematic review and Meta-analysis. PLoS One.

[4] Khan A, Brown WA (2015). Antidepressants versus placebo in major depression: an overview. World psychiatry : official journal of the World Psychiatric Association (WPA).

[5] McGrath PJ, Stewart JW, Quitkin FM (2006). Predictors of relapse in a prospective study of fluoxetine treatment of major depression. Am J Psychiatry.

[6] Khan A, Warner HA, Brown WA (2000). Symptom reduction and suicide risk in patients treated with placebo in antidepressant clinical trials. Arch Gen Psychiatry.

[7] Adjibade M, Lemogne C, Touvier M (2019). The inflammatory potential of the diet is directly associated with incident depressive symptoms among French adults. J Nutr.

[8] Bergmans RS, Malecki KM (2017). The association of dietary inflammatory potential with depression and mental well-being among U.S. adults. Prev Med.

[9] Godos J, Currenti W, Angelino D (2020). Diet and mental health: review of the recent updates on molecular mechanisms. Antioxidants.

[10] Lee, C.H., Giuliani, F. The Role of Inflammation in Depression and Fatigue. Front Immunol. 2019;10:1696. Published 2019 Jul 19. doi:10.3389/fimmu.2019.01696 10.3389/fimmu.2019.01696 PMC6658985 31379879

[11] Fountoulakis KN, Gonda X, Rihmer Z (2008). Revisiting the dexamethasone suppression test in unipolar major depression: an exploratory study. Ann General Psychiatry.

[12] Gałecki P, Talarowska M (2018). Inflammatory theory of depression. Psychiatr Pol.

[13] Lamers F, Milaneschi Y, Smit JH, Schoevers RA, Wittenberg G, Penninx BWJH (2019). Longitudinal association between depression and inflammatory markers: results from the Netherlands study of depression and anxiety. Biol Psychiatry.

[14] Valkanova V, Ebmeier KP, Allan CL (2013). CRP, IL-6 and depression: a systematic review and meta-analysis of longitudinal studies. J Affect Disord.

[15] Osimo EF, Pillinger T, Rodriguez IM, Khandaker GM, Pariante CM, Howes OD (2020). Inflammatory markers in depression: a meta-analysis of mean differences and variability in 5,166 patients and 5,083 controls. Brain Behav Immun.

[16] Tabung FK, Giovannucci EL, Giulianini F (2018). An empirical dietary inflammatory pattern score is associated with circulating inflammatory biomarkers in a multi-ethnic population of postmenopausal women in the United States. J Nutr.

[17] Popa TA, Ladea M (2012). Nutrition and depression at the forefront of progress. J Med Life.

[18] Ljungberg T, Bondza E, Lethin C (2020). Evidence of the importance of dietary habits regarding depressive symptoms and depression. Int J Environ Res Public Health.

[19] Głąbska D, Guzek D, Groele B, Gutkowska K. Fruit and vegetable intake and mental health in adults: a systematic review. Nutrients. 2020;12(1). 10.3390/nu12010115 PMC7019743 31906271

[20] Lai JS, Hiles S, Bisquera A, Hure AJ, McEvoy M, Attia J (2013). A systematic review and meta-analysis of dietary patterns and depression in community-dwelling adults. Am J Clin Nutr.

[21] Opie RS, Ball K, Abbott G, Crawford D, Teychenne M, McNaughton SA. Adherence to the Australian dietary guidelines and development of depressive symptoms at 5 years follow-up amongst women in the READI cohort study. Nutr J. 2020;19(1). 10.1186/s12937-020-00540-0 PMC7149932 32276594

[22] Tabung FK, Smith-Warner SA, Chavarro JE (2016). Development and validation of an empirical dietary inflammatory index. J Nutr.

[23] Tabung FK, Smith-Warner SA, Chavarro JE (2017). An empirical dietary inflammatory pattern score enhances prediction of circulating inflammatory biomarkers in adults. J Nutr.

[24] Jain FA, Walsh RN, Eisendrath SJ, Christensen S, Rael Cahn B (2015). Critical analysis of the efficacy of meditation therapies for acute and subacute phase treatment of depressive disorders: a systematic review. Psychosomatics..

[25] Kanauchi M, Shibata M, Iwamura M (2019). A novel dietary inflammatory index reflecting for inflammatory ageing: technical note. Ann Med Surg.

[26] Kroenke K, Spitzer RL (2002). The PHQ-9: a new depression and diagnostic severity measure. Psychiatr Ann.

[27] Kroenke K, Spitzer RL, Williams JB (2001). The PHQ-9: validity of a brief depression severity measure. J Gen Intern Med.

[28] Tolkien K, Bradburn S, Murgatroyd C (2019). An anti-inflammatory diet as a potential intervention for depressive disorders: a systematic review and meta-analysis. Clin Nutr.

[29] Wang J, Zhou Y, Chen K (2018). Dietary inflammatory index and depression: a meta-analysis. Public Health Nutr.

[30] Tiu RT (2017). The microbiome as a novel paradigm in studying stress and mental health. Am Psychol.

[31] Akbaraly TN, Kerleau C, Wyart M (2016). Dietary inflammatory index and recurrence of depressive symptoms: results from the Whitehall II study. Clin Psychol Sci.

[32] Parletta N, Zarnowiecki D, Cho J (2017). A Mediterranean-style dietary intervention supplemented with fish oil improves diet quality and mental health in people with depression: a randomized controlled trial (HELFIMED). Nutr Neurosci.

[33] Haftenberger M, Heuer T, Heidemann C, Kube F, Krems C, Mensink GB (2010). Relative validation of a food frequency questionnaire for national health and nutrition monitoring. Nutr J.

[34] Sahebi A, Nejati-Zarnaqi B, Moayedi S, Yousefi K, Torres M, Golitaleb M (2021). The prevalence of anxiety and depression among healthcare workers during the COVID-19 pandemic: an umbrella review of meta-analyses. Prog Neuro-Psychopharmacol Biol Psychiatry.

