# Correction: Pro-inflammatory diet and depressive symptoms in the healthcare setting

**DOI:** 10.1186/s12888-022-04489-8

**Published:** 2022-12-30

**Authors:** Rachel Belliveau, Sydney Horton, Courtney Hereford, Lance Ridpath, Robert Foster, Emily Boothe

**Affiliations:** 1 University of North Carolina Health Care, 2201 S Sterling St, Morganton, NC USA; 2 University of Texas Health Science Center at Tyler, 11937 US-271, Tyler, TX USA; 3 Center for Rural and Community Health, West Virginia School of Osteopathic Medicine, 400 N Lee St, Lewisburg, WV USA; 4 Institutional Research Assessment Educational Development, West Virginia School of Osteopathic Medicine, 400 N Lee St, Lewisburg, WV USA; 5 West Virginia School of Osteopathic Medicine, 400 N Lee St, Lewisburg, WV USA; 6 Department of Psychiatry, Princeton Community Hospital, 122 12th St, Princeton, WV USA

**Correction: BMC Psychiatry 22, 125 (2022)**

**https://doi.org/10.1186/s12888-022-03771-z**

Following publication of the original article [1], the authors identified an error in Table 3. The correct table is given below.

**Table 3:** EDII score compared to PHQ-9 score. Low EDII score correlates to a lower PHQ-9 score; high EDII score correlates to a higher PHQ-9 score

EDII Score	PHQ-9 Score		
	N	Mean	SD
*Low (− 12 to − 2)*	166	5.83	5.42
*Moderate (− 1 to + 1)*	306	6.60	5.50
*High (+ 2 to + 10)*	159	8.45	5.78

Key: *N* Population, *SD* Standard deviation

The original article [1] has been corrected.
